# iMet-Q: A User-Friendly Tool for Label-Free Metabolomics Quantitation Using Dynamic Peak-Width Determination

**DOI:** 10.1371/journal.pone.0146112

**Published:** 2016-01-19

**Authors:** Hui-Yin Chang, Ching-Tai Chen, T. Mamie Lih, Ke-Shiuan Lynn, Chiun-Gung Juo, Wen-Lian Hsu, Ting-Yi Sung

**Affiliations:** 1 Bioinformatics Program, Taiwan International Graduate Program, Academia Sinica, Taipei 11529, Taiwan; 2 Institute of Biomedical Informatics, National Yang-Ming University, Taipei 11221, Taiwan; 3 Institute of Information Science, Academia Sinica, Taipei 11529, Taiwan; 4 Department of Mathematics, Fu Jen Catholic University, New Taipei City 24205, Taiwan; 5 Molecular Medicine Research Center, Chang Gung University, Taoyuan 33302, Taiwan; Imperial College London, UNITED KINGDOM

## Abstract

Efficient and accurate quantitation of metabolites from LC-MS data has become an important topic. Here we present an automated tool, called iMet-Q (intelligent Metabolomic Quantitation), for label-free metabolomics quantitation from high-throughput MS1 data. By performing peak detection and peak alignment, iMet-Q provides a summary of quantitation results and reports ion abundance at both replicate level and sample level. Furthermore, it gives the charge states and isotope ratios of detected metabolite peaks to facilitate metabolite identification. An in-house standard mixture and a public Arabidopsis metabolome data set were analyzed by iMet-Q. Three public quantitation tools, including XCMS, MetAlign, and MZmine 2, were used for performance comparison. From the mixture data set, seven standard metabolites were detected by the four quantitation tools, for which iMet-Q had a smaller quantitation error of 12% in both profile and centroid data sets. Our tool also correctly determined the charge states of seven standard metabolites. By searching the mass values for those standard metabolites against Human Metabolome Database, we obtained a total of 183 metabolite candidates. With the isotope ratios calculated by iMet-Q, 49% (89 out of 183) metabolite candidates were filtered out. From the public Arabidopsis data set reported with two internal standards and 167 elucidated metabolites, iMet-Q detected all of the peaks corresponding to the internal standards and 167 metabolites. Meanwhile, our tool had small abundance variation (≤0.19) when quantifying the two internal standards and had higher abundance correlation (≥0.92) when quantifying the 167 metabolites. iMet-Q provides user-friendly interfaces and is publicly available for download at http://ms.iis.sinica.edu.tw/comics/Software_iMet-Q.html.

## Introduction

Unbiased quantitation and identification of small-molecule metabolites in a biological system is important because metabolites serve as direct signatures of biochemical activity, making them relatively easier to correlate with disease phenotype and more suitable for clinical diagnostics [1–8]. Liquid chromatography coupled with mass spectrometry (LC-MS) has become a conventional platform for analyzing metabolites across a large number of biological samples because of its ability to measure thousands of metabolites in a short period of time [2, 5, 9–13]. However, it is a time-consuming process to manually calculate the abundances of thousands of metabolites from large-scale LC-MS data, and thus developing a software package to automatically quantify LC-MS metabolomics data becomes essential.

Many commercial software programs have been developed by instrument vendors, such as Progenesis QI (Waters), MassHunter (Agilent), MultiQuant (Sciex), and Xcalibur (Thermo). But they usually accept data formats specific to their own instruments, and thus limit the possibility of integrating metabolite analyses across different analytical platforms [14–17]. Additionally, data processing algorithms implemented in these programs are mostly unavailable. Publicly available quantitation tools, on the other hand, accept multiple input data formats and provide description of their computational algorithms. For example, XCMS [18], MetAlign [19] and MZmine 2 [20] accept input formats such as mzXML, mzData, and netCDF. XCMS, implemented in R, C, and C++ programming language, provides format conversion, noise filtering, peak detection, non-linear spectral alignment algorithms, and statistical analysis of LC-MS data. MetAlign, an open interface-driven tool implemented in C++ programming language, provides format conversion, mass calculation, baseline correction, peak-picking, saturation and mass peak artifact filtering, as well as data set alignment. MZmine 2, a modular-based tool implemented in Java programming language, provides raw data import, raw data processing, peak detection, peak list alignment, normalization, visualization, peak identification, and statistical analysis.

However, some concerns of these public tools have been reported. For instance, some tools combine binning with model fitting or use slope calculation approaches to perform peak detection, but these approaches may have low sensitivity on peak picking because the peak shapes vary greatly from one to another [21, 22]. Due to different peak detection and peak alignment algorithms provided in each tool, it is difficult for users to optimize the algorithm-related parameters, which very likely affect the quantitation results [23, 24]. On the other hand, most of the tools do not provide deisotoping, and thus users have to apply additional packages, such as CAMERA [25], to perform isotope pattern assembly [21].

To rectify the above concerns, we develop a quantitation tool, called iMet-Q (intelligent Metabolomic Quantitation), written in C# programming language, which provides highly accurate quantitation and user-friendly graphical interfaces. For peak detection, iMet-Q requires one input parameter, i.e., mzWidth (defined as the width of a peak in m/z dimension), and calculates the full width at half maximum (FWHM) of a peak to dynamically determine its chromatographic width so that the peak boundary can be automatically determined (i.e., without requiring parameter input). In addition, our tool determines the charge state of a peak by calculating the similarity between the peak shape of monoisotope and the peak shape of the second isotope peak. Once the charge state is determined, iMet-Q calculates its isotope ratio (i.e., the ratio of the second isotope abundance to the monoisotope abundance) to facilitate metabolite identification. Since metabolites usually have smaller retention time drifts across LC-MS technical replicates, iMet-Q first aligns peaks across different LC-MS replicates of a sample and then across different samples. In each alignment, LOESS regression algorithm [26, 27] is applied to adjust peak retention time so that peaks in different LC-MS replicates/samples can be aligned according to their *m/z* and adjusted retention time values.

We used two metabolite data sets, an in-house standard mixture and a public Arabidopsis metabolome data set [28], to evaluate the performance of iMet-Q and compare it with three public quantitation tools, including XCMS, MetAlign, and MZmine2. Since the standard mixture was acquired in profile mode, we evaluated the performance of the four tools in both profile and centroid data sets, in which the centroid data set was converted from the profile data set by the centroiding function of ProteoWizard [29]. As a result, iMet-Q had small quantitation error of 12% in both profile and centroid data sets. In addition, our tool correctly determined the charge state of seven standard metabolites, removed their isotope peaks, and calculated their isotope ratios. With the isotope ratios, we successfully filtered out 49% (89 out of 183) metabolite candidates when using mass values of these standard metabolites to search in Human Metabolome Database (HMDB) [30]. For the public Arabidopsis metabolome data set reported with two internal standards and 167 elucidated metabolites, iMet-Q detected the internal standards from all replicates and samples. In addition, all of the 167 metabolites were also detected by iMet-Q, whereas 103 (62%), 145 (87%), and 129 (77%) elucidated metabolites were detected by XCMS, MetAlign, and MZmine 2, respectively. Comparing with the three quantitation tools, iMet-Q had relatively small abundance variations (≤0.19) when quantifying the internal standards, and had higher abundance correlation (≥0.92) when quantifying the 167 metabolites across replicates. All data underlying the manuscript are fully available without restriction. The source code of iMet-Q is freely available on SourceForge (http://sourceforge.net/projects/imet-q/?source=navbar) under the license of GPL2.

## Materials and Methods

### Experiment on standard metabolite mixture

#### Chemicals

All chemicals and solvents were purchased from Sigma Aldrich (St. Louis, MO, USA). The chemicals were all of analytical grade. Water containing 0.1% formic acid (solvent A), and acetonitrile containing 0.1% formic acid (solvent B) were of CHROMASOLV grade.

#### Sample preparation

Stoke solution of metabolites was prepared as 1 mg/mL of 80% MeOH solution with 0.1% formic acid. Two metabolite mixtures, Sample 1 and Sample 2, were prepared by adding seven standard metabolites with different amount into two 1.7 mL vials. These standard metabolites were L-histidine (3 μL in Sample 1, 6μL in Sample 2), L-carnosine (50μL in Sample 1, 50μL in Sample 2), creatine (6μL in Sample 1, 6μL in Sample 2), caffeine (6μL in Sample 1, 6μL in Sample 2), hippuric acid (5μL in Sample 1, 25μL in Sample 2), glycocholic acid (2μL in Sample 1, 10μL in Sample 2), and cholic acid (2μL in Sample 1, 6μL in Sample 2). The solutions were dried with SpeedVac and re-dissolved by 100 L of MeOH and water (2:1, v/v) with 0.1% formic acid.

#### UPLC/LTQ-Orbitrap MS

A UPLC system (Acquity, Waters, Milford, MA, USA) equipped with a C18 reversed-phase column (2.1 × 100 mm, 1.8 m, HSS-T3; Waters, Milford, MA, USA) was coupled with a LTQ-Orbitrap mass spectrometer (Thermo Scientific, San Jose, CA, USA) with an orthogonal electrospray ionization (ESI) source. The initial flow rate was 0.1 mL/min of 99% solvent A (0.1% formic acid) and 1% solvent B (acetonitrile with 0.1% formic acid). A volume of 1μL of Sample 1 and Sample 2 were sequentially injected, and 6 technical replicates were conducted in profile mode on each standard mixture. After injection, solvent B was maintained at 1% for 5 min, then increased to 50% during a time span of 9 min, then to 90% over 6 min, and finally to 99% over a period of 12 min after which this percent composition was held for 1 min. The flow rate was changed to 0.5 mL/min, and after 5 min reduced to 0.1mL/min. After 0.1 min, solvent B was reduced back to 1% and held at this percentage for 7 min.

The mass spectrometer was controlled by using Xcalibur 2.0.7 software (Thermo Scientific) and operated in positive ion mode on the *m/z* range from 120 to 1,000 with 60,000 resolution at *m/z* 400. The source and capillary voltages were set to 4500 and 35 V, and the temperatures of the capillary and the APCI vaporizer were set to 275°C and 150°C. Internal calibration was performed using the ion signal of dioctyl phthalate at *m/z* 391.28 as a lock mass.

### Public Arabidopsis metabolome data set

A public Arabidopsis metabolome data set of 36 samples acquired by LC-ESI-Q-TOF/MS (HPLC: Waters Acquity UPLC system; MS: Waters Q-TOF Premier) in positive- and negative-ion modes, with four technical replicates on each sample, was downloaded from the website of PRIMe (http://prime.psc.riken.jp/). All replicates of the Arabidopsis metabolome data set were acquired in centroid mode. Two internal standards, lidocaine (*m/z* = 235 [M+H]+, eluted at 4.19 min in the positive-ion mode data) and camphor-10-sulfonic acid (*m/z* = 231 [M-H]-, eluted at 3.84min in the negative-ion mode data) were spiked in each sample with the same amount of 0.5 mg/L for abundance normalization and 167 metabolites were elucidated from this data set [31, 32].

### Data processing and software parameter settings

Data processing was performed on a Microsoft Windows 7 computer (x64 edition service pack 1 with an Intel Xeon E5-2630 processor, 16 GB RAM and 500GB hard disk drive). All raw data files of the standard mixture experiment and the public data set were converted into mzXML format using ProteoWizard. The acquired profile-mode data of standard mixture experiment was converted into two data sets, where one data set was directly converted to mzXML files (called *profile data set* for convenience) and the other was converted to mzXML files with an additional centroiding procedure in ProteoWizard (called *centroid data set* for convenience). Since the public data was acquired in centroid mode, no additional centroiding procedure in ProteoWizard was performed.

Both profile and centroid data sets of standard mixture were processed by four tools. iMet-Q automatically skipped centroiding step when processing the centroid data set and used the following parameter setting: mzWidth = 0.02, mzTol = 0.02, and rtTol = 0.2 for both data sets. For XCMS, peaks in the profile and centroid data sets were first detected using *matchedFilter* and c*entWave* function, respectively. Then the *rector*.*loess* and *group*.*density* functions were then applied to align the detected peaks across samples. An additional *fillPeaks* fuction was performed for the recovery of missing peaks as suggested by the package vignette of XCMS. To optimize the XCMS parameters, 42 parameter combinations were examined using the standard metabolites in the standard mixture as benchmarks. The examination results of the 42 parameter combinations are listed in S1 and S2 Tables, and the quantitation results generated using those parameter combinations are provided in S1 and S2 Files. The optimized XCMS parameters on peak picking are: method = matchedFilter, fwhm = 30, snthresh = 10 for profile dataset; and method = centWave, ppm = 45, snthresh = 10, peakwidth = (5,20) for centroid dataset. The optimized XCMS parameters for both profile and centroid data sets on retention time correction and peak grouping are: method = loess, span = .67; and method = "density", mzwid = 0.03, bw = 3, minfrac = 0, minsamp = 0. MetAlign only accepts and processes the centroid data set. To run MetAlign, we selected the data type of accurate mass data with mass resolution of 10,000 and applied the default settings for the remaining parameters. In MZmine 2, the *exact mass detector* function and the *centroid mass detector* function were first applied to process the profile and centroid data sets, respectively, both with a noise level of 1,000,000. Then the following functions were applied using the same parameters to process both data sets. The chromatogram builder was used with the minimum time span, minimum height, and *m/z* tolerance set to be 0, 0 and 0.02, respectively. For chromatogram deconvolution, baseline cut-off algorithm was used to detect peaks, where the minimum peak height, peak duration length, and baseline level were 1,000,000, 0–2 min, and 1,000,000, respectively. The *m/z* and retention time tolerances in both isotopic peaks grouper and peak alignment were 0.02 and 0.2 min, respectively.

Public Arabidopsis data set was processed by iMet-Q with mzWidth, mzTol, and rtTol of 0.03, 0.03, and 0.16 min, respectively. We used the XCMS c*entWave* function for peak detection, *rector*.*loess* and *group*.*density* functions for peak alignment, and *fillPeaks* function for the recovery of missing peaks. To optimize XCMS parameters on the public Arabidopsis positive- and negative-mode data sets, 37 parameter combinations were examined on each set and the parameter combination achieving the best quantitation performance was selected. The examination results of the parameter combinations are listed in S3 and S4 Tables, and the quantitation results generated using those parameter combinations are provided in S3 and S4 Files. Both data sets used centWave method in peak picking and the optimized XCMS parameters for Arabidopsis positive- and negative-mode datasets are: ppm = 30, snthresh = 10, peakwidth = (25, 40); and ppm = 20, snthresh = 10, peakwidth = (5, 20), respectively. Next, in retention time correction and peak grouping, the optimized XCMS parameters for both profile and centroid data sets are: method = loess, span = .67; and method = "density", mzwid = 0.03, bw = 3, minfrac = 0, minsamp = 0. For MetAlign, we selected the data type of accurate mass data with mass resolution of 9,000 and applied the following parameter settings for data processing: maximum amplitude = 10,000, peak slope factor = 1, peak threshold factor = 6, average peak width at half weight = 8, maximum shift per scan = 35, select min nr per peak set = 4, and scaling options = none. In MZmine 2, we used the centroid mass detector with a noise level of 6. The minimum time span, minimum height, and *m/z* tolerance in the chromatogram builder were set to be 0.1 min, 6 and 0.03, respectively. For chromatogram deconvolution, baseline cut-off algorithm was used to detect peaks, where the minimum peak height, peak duration length, and baseline level were 6, 0–1.6 (min), and 6, respectively. The *m/z* and retention time tolerances in both isotopic peaks grouper and peak alignment were 0.03 and 0.16 min, respectively. Detected peaks by each tool were retained in the quantitation results if their retention time values were within the effective retention time range (i.e., 0.85 to 12 min) and the peaks were detected in at least one sample.

### Robust algorithms for metabolite quantitation

**Fig 1:**
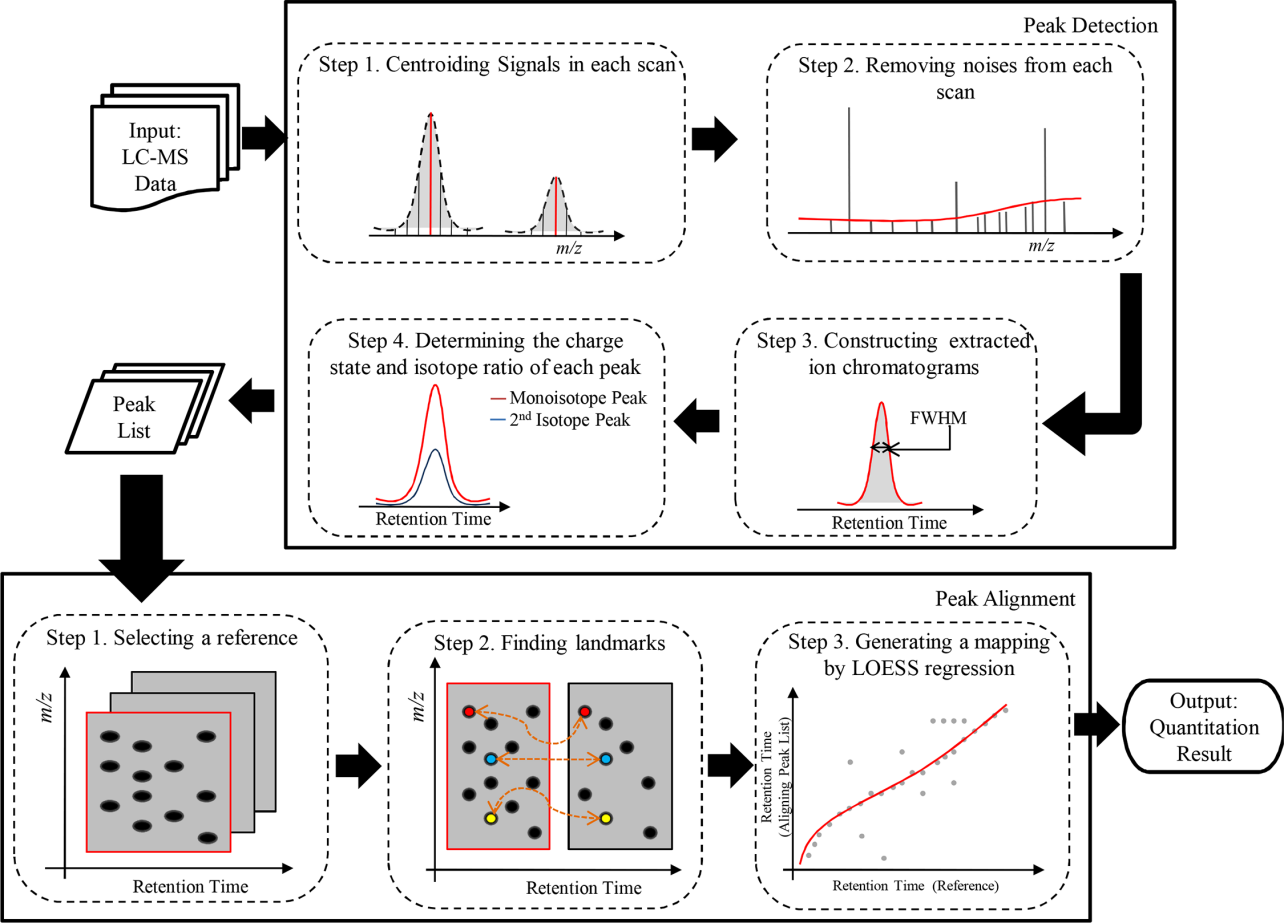
Schematic depiction of iMet-Q workflow for peak detection and peak alignment.

In order to process data from various mass spectrometers, iMet-Q accepts input files in netCDF, mzXML, and mzML formats, which can be conveniently converted from raw data by existing converters. Also, it accepts profile-mode and centroid-mode data. The workflow of iMet-Q contains two main tasks, peak detection and peak alignment, as depicted in Fig 1. Performing peak detection on each input file generates a peak list that reports the *m/z*, retention time, charge state, isotope ratio, and abundance of detected peaks. To compare compounds across different samples, iMet-Q aligns the detected peaks in the peak lists according to their *m/z* and retention time. The detailed procedures of peak detection, peak alignment and quantitation result generation are described below.

### Peak Detection

#### Centroiding signals in each scan

Centroiding, which can be performed during or after data acquisition, is a common first step for processing metabolomics data so that the data size can be greatly reduced. It aims at combining multiple signals from the same compound into a single one with an *m/z* and intensity value [32]. If the input files have already been centroided, iMet-Q automatically skips this step. The detailed algorithm description is listed in S1 Section.

#### Removing noises from each scan

Due to nonspecific nature of ESI, a great portion of signals in the spectra could be noise [33]. It is essential to remove these noises to increase the effectiveness of peak detection. Instead of determining a fixed baseline for each spectrum, iMet-Q equally divides a scan into *s* segments (*s* = 10 by default) with an overlap of 20% to avoid borderline issues and defines the average intensity in a segment as its noise level. Then, for each segment, signals with S/N less than a specific threshold (3 by default) are removed from the scan, where S is the signal intensity and N is the noise level in the segment. The number of segments and the S/N threshold are user-adjustable.

#### Constructing extracted ion chromatograms (EIC) with dynamically determining peak width

**Fig 2:**
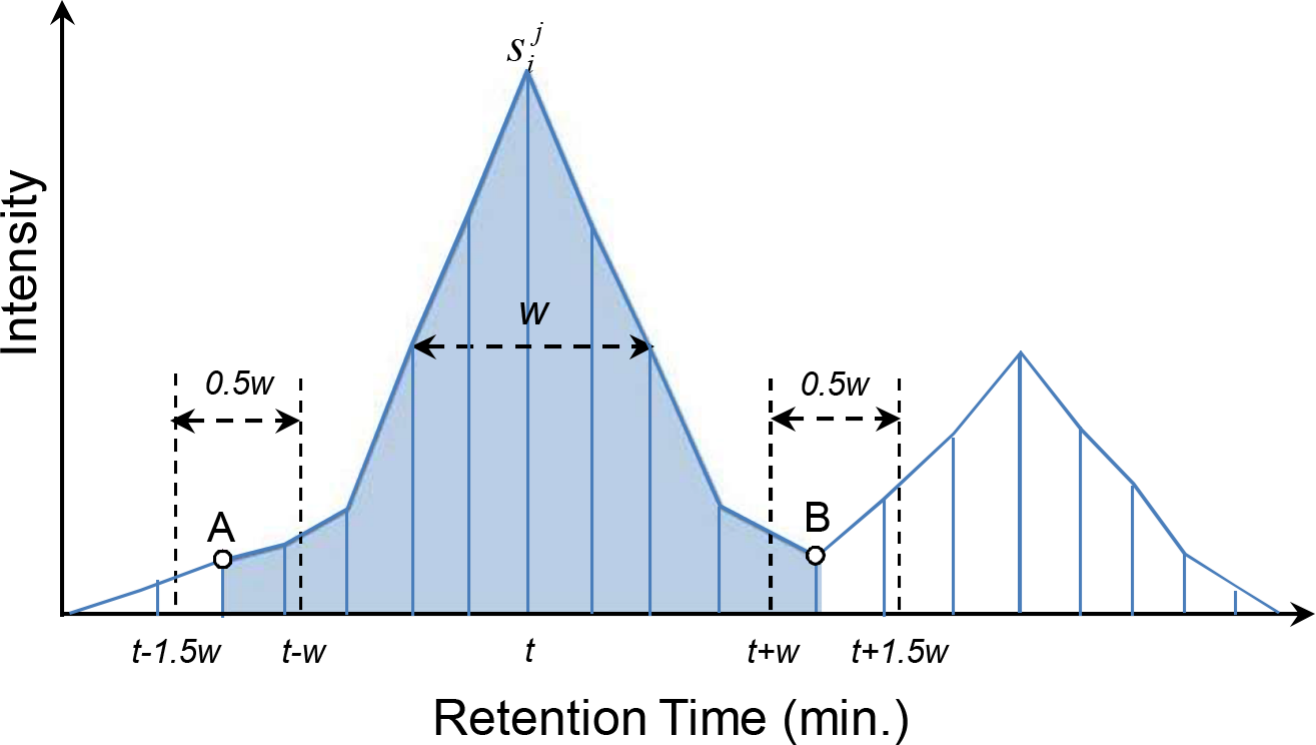
A cartoon for the illustration of constructing extracted ion chromatograms. The blue straight lines represent the clustered signals, *w* and *t* are the FWHM and retention time of $s_{i}^{j}$, respectively. Signal A and B are determined as the boundaries of the EIC, and the area in light blue color is the abundance.

After processing signals in each scan, iMet-Q uses a two-stage algorithm to construct the extracted ion chromatogram (EIC) of a compound. The two-stage algorithm includes signal clustering and EIC boundary determination. Let $S=\{\{s_{1}^{1},s_{2}^{1},\ldots ,s_{n_{1}}^{1}\},\{s_{1}^{2},s_{2}^{2},\ldots ,s_{n_{2}}^{2}\},\ldots ,\{s_{1}^{m},s_{2}^{m},\ldots ,s_{n_{k}}^{m}\}\}$ be the list of signals in all scans, where *m* is the total number of scans and *n*_*k*_ is the number of signals in the *k*^th^ scan. In signal clustering, starting from the most intensive signal, say $s_{i}^{j}$, among all scans in *S*, iMet-Q clusters signals in *S* with $s_{i}^{j}$ if the *m/z* differences between the signals and $s_{i}^{j}$ are within the given tolerance *d*, i.e., mzWidth in iMet-Q. A cluster with at least three signals is further processed. In the step of EIC boundary determination, iMet-Q automatically calculates FWHM and then dynamically determines the boundaries using FWHM. To be specific, for the most intensive signal in a cluster, say $s_{i}^{j}$, with retention time *t*, the boundaries of the EIC are initially determined as *t* ± *w*, where *w* is the FWHM. To adapt to possible tailing effect of an EIC, the boundaries are further extended by a tolerance at most 0.5*w* and are set to the farthest signal or a signal with local-minimum intensity in this range (as shown in Fig 2). Then the trapezoid areas between any two adjacent signals of the EIC are summed up as the abundance. The two-stage algorithm is iteratively performed on the remaining unclustered signals to construct EICs until all signals in *S* are processed. In the following subsections, we term the constructed EICs as peaks for convenience. Furthermore, each peak is represented by its apex, i.e., using the m/z, retention time of the apex signal, and the associated abundance is recorded.

#### Determining the charge state and isotope ratio of peaks

In order to determine the charge state and isotope ratio, iMet-Q first constructs an isotope envelope for a peak. Starting from the most intensive peak, *P*_*1*_, in the current peak list, our tool groups it with all of its neighboring peaks having larger *m/z* values and decreasing intensities, i.e., forming an isotope group. If the *m/z* distance between the apex of any two peaks in the isotope group equals to 1/*z* (*z* can be 1~4), the group is considered as a possible isotope envelope, where *P*_*1*_ and the peak adjacent to *P*_*1*_ are considered as the monoisotope peak and the second isotope peak, respectively. Then, to validate the correctness of the isotope envelope, iMet-Q calculates the peak shape similarity between the monoisotope peak and the second isotope peak by using the dot product as shown below:

$$Similarity\;=\frac{\sum_{i=1}^{n}x_{i}y_{i}}{\sqrt{\sum_{i=1}^{n}x_{i}^{2}}\sqrt{\sum_{i=1}^{n}y_{i}^{2}}}$$ (1)

where *n* is the number of scans that both the monoisotope peak and the second isotope peak co-occur, and *x*_*i*_ and *y*_*i*_ are their respective signal intensities in the *i*^th^ scan. If the peak shape similarity between the two peaks is above a user-defined threshold (0.8 by default), we consider that all of the peaks in the isotope group form an isotope envelope and the charge state of *P*_1_ is determined as *z*. Moreover, the isotope ratio of *P*_*1*_ is calculated as the abundance of the second isotope peak divided by the abundance of *P*_*1*_, and the peaks in the isotope group are removed from further processing. Otherwise, the peaks do not form an isotope envelope and the charge state and isotope ratio of *P*_*1*_ are denoted as 0. The charge state determination procedure is repeatedly performed until all peaks are processed.

By performing the above peak detection procedures, iMet-Q generates a peak list with charge state and isotope ratio information for each input replicate file. Note that the isotope peaks except the monoisotope in the confirmed isotope envelopes will not be reported in the peak list.

### Peak Alignment

For an input data set with multiple samples (each probably with multiple technical replicates), iMet-Q performs the same algorithm for pair-wise alignment within a sample (i.e., aligning replicates) and then across samples. The algorithm is described as follows.

iMet-Q first selects the peak list that contains the largest number of detected peaks as the reference list and aligns other peak lists to it. For a pair of reference list and aligning peak list, iMet-Q uses peaks detected in both lists with their *m/z* and retention time differences within user-defined tolerances (i.e., mzTol and rtTol in iMet-Q) as landmarks [34]. Given the retention time of landmarks in the reference list and in the aligning peak list as vectors A and B, respectively, iMet-Q uses LOESS regression [26, 27] to generate a mapping between A and B with span of 20% and weight of 1 since LOESS regression combines multiple linear least square regression models which fit localized subsets of the data. The retention time of peaks, which are not landmarks, in either peak list are adjusted according to the LOESS regression model. iMet-Q then aligns the peaks in both peak lists that satisfy user-defined *m/z* tolerance and retention time tolerance based on adjusted retention time.

Repeating the above process for all peak lists, peaks can be aligned across replicates. Since peaks aligned from different replicates in a sample may have slightly different values of *m/z* and retention time, we use the *median* of their *m/z* and retention time as the representative peak in each sample. Then the same algorithm is applied to align all of the representative peaks across samples.

### Generating output of quantitation results

In addition to viewing quantitation results in the main interface, iMet-Q reports the medians of *m/z*, retention time and abundance of the peaks in all replicates of all samples. For the charge state of an aligned peak, iMet-Q displays the charge state that is detected in most replicates. For the isotope ratio, iMet-Q collects the isotope ratios corresponding to the displayed charge state and reports the median as the isotope ratio. Quantitation results can be exported in.csv and.txt formats by users to conduct further analyses.

## Results and Discussion

We evaluated the performance of iMet-Q in metabolite quantitation by comparing the accuracy of detecting metabolites and the abundance consistency, calculated by Pearson product-moment correlation coefficient (PPMCC), of detected metabolites among replicates with XCMS, MetAlign, and MZmine 2 using samples ranging from a small standard data set (seven standard metabolites) to a complex biological data set (public Arabidopsis data set). We also demonstrated the ability of iMet-Q on charge state determination and isotope ratio calculation.

### Performance evaluation on a standard metabolite mixture data

To demonstrate the ability of iMet-Q, we used a standard metabolite mixture in which seven standard metabolites with different sample concentration in two samples were included for evaluation. The sample ratio, defined as the detected abundance of a standard metabolite in Sample 1 divided by that in Sample 2, is calculated for each of the seven metabolites in the standard metabolite mixture for the four quantitation tools. Both profile and centroid data sets generated from the raw data were processed by iMet-Q, XCMS, MetAlign, and MZmine 2, respectively. Algorithms and parameter settings used in these tools were described in the subsection of *Data Processing and Software Parameter Settings*. Table 1 shows the quantitation errors, which is defined as the difference between theoretical and calculated sample ratios divided by the theoretical sample ratios, of the seven standard metabolites calculated by the four quantitation tools. Detailed abundances and sample ratios calculated by the four tools are listed in S5 Table.

**Table 1:** The quantitation error (%) of seven standard metabolites calculated by iMet-Q, XCMS, MetAlign, and MZmine 2.

		Quantitation error (%)^a^							
		iMet-Q		XCMS		MetAlign		MZmine 2	
Standard Compound	Theoretical Sample Ratio	Profile	Centroid	Profile	Centroid	Profile	Centroid	Profile	Centroid
L-hisitidine	0.50	14	14	14	2	-	12	14	12
L-Carnosine	1.00	9	8	7	50	-	11	8	8
Creatine	1.00	12	12	12	18	-	12	12	11
Caffine	1.00	15	16	14	2	-	14	15	16
Hippuric acid	0.20	10	10	10	105	-	5	15	20
Glycocholic acid	0.20	20	20	20	20	-	30	5	5
Cholic acid	0.33	6	6	6	24	-	12	24	24
	Average	12	12	12	32	-	14	13	14

^a^Quantitation error (%) = (|Theoretical Sample Ratio–Calculated Sample Ratio|) x100/ Theoretical Sample Ratio.

As shown in Table 1, iMet-Q, XCMS, and MZmine 2 had the average quantitation error of 12%, 12% and 13% on the profile data set, and 12%, 32% and 14% on the centroid data set. MetAlign did not process the profile data set and had the average quantitation error of 14% on the centroid data set. The results showed that iMet-Q achieved the smallest quantitation errors and also had the same average quantitation error of 12% in both profile and centroid data set, suggesting the robustness of iMet-Q on processing input files in both data type.

In addition to accurate quantitation, iMet-Q determined the charge state and isotope ratio of detected peaks. In order to evaluate the charge state determined by iMet-Q, we used CAMERA to annotate charge states and isotope peaks of the standard metabolites. Charge states and isotope peaks of the seven standard metabolites in both profile and centroid data sets annotated by iMet-Q were overall identical with CAMERA’s annotation.

To demonstrate possible usefulness of isotope ratio information, we searched the mass of seven standard metabolites against HMDB [30] with mass tolerance of ±5 ppm and adduct type of unknown (assuming a user has little information on the spectra). As a result, 183 metabolites candidates were obtained. Using iMet-Q’s calculated isotope ratios (the isotope ratio tolerance is ±0.02) to filter out unnecessary metabolite candidates, we reduced 49% (89 out of 183) metabolite candidates (S6 Table). The remaining metabolite candidates could not be filtered out by isotope ratios because they have the same chemical formula (i.e., identical theoretical isotope ratios).

### Performance evaluation on a public Arabidopsis metabolome data

The public Arabidopsis data of 36 distinct samples collected from eight different plant classes (classified by Matsuda *et al*.) was used to demonstrate iMet-Q’s performance on quantifying complex biological samples. Each sample contained two internal standards, one for positive- and the other for negative-ion modes, and 167 metabolites (71 detected only in positive-ion mode, 39 only in negative-ion mode, and 57 in both modes) were elucidated from the data set [28]. The performance of four tools were evaluated in terms of the accuracy on detecting the two internal standards and 167 elucidated metabolites, and the ability of properly clustering 36 samples into eight plant classes.

Table 2 shows the reproducibility and normalized abundances of two internal standards detected by the four quantitation tools, where iMet-Q, XCMS, and MetAlign detected all the internal standards from each replicate, achieving 100% reproducibility, slightly better than MZmine 2. Furthermore, iMet-Q reported normalized abundances of 1±0.19 and 1±0.18 for the two standard metabolites, comparable to the best 1±0.18 reported by XCMS and MZmine 2 for Lidocaine and the best 1±0.17 reported by XCMS for Camphor-10-sulfonic acid.

**Table 2:** The reproducibility (Rep.) and normalized abundance (Abund.) of two internal standards detected by four quantitation tools.

	Lidocaine		Camphor-10-sulfonic acid	
	Rep. (%)	Abund.	Rep. (%)	Abund.
iMet-Q	100	1±0.19	100	1±0.18
XCMS	100	1±0.18	100	1±0.17
MetAlign	100	1±0.20	100	1±0.22
MZmine 2	100	1±0.18	85	1±0.46

**Fig 3:**
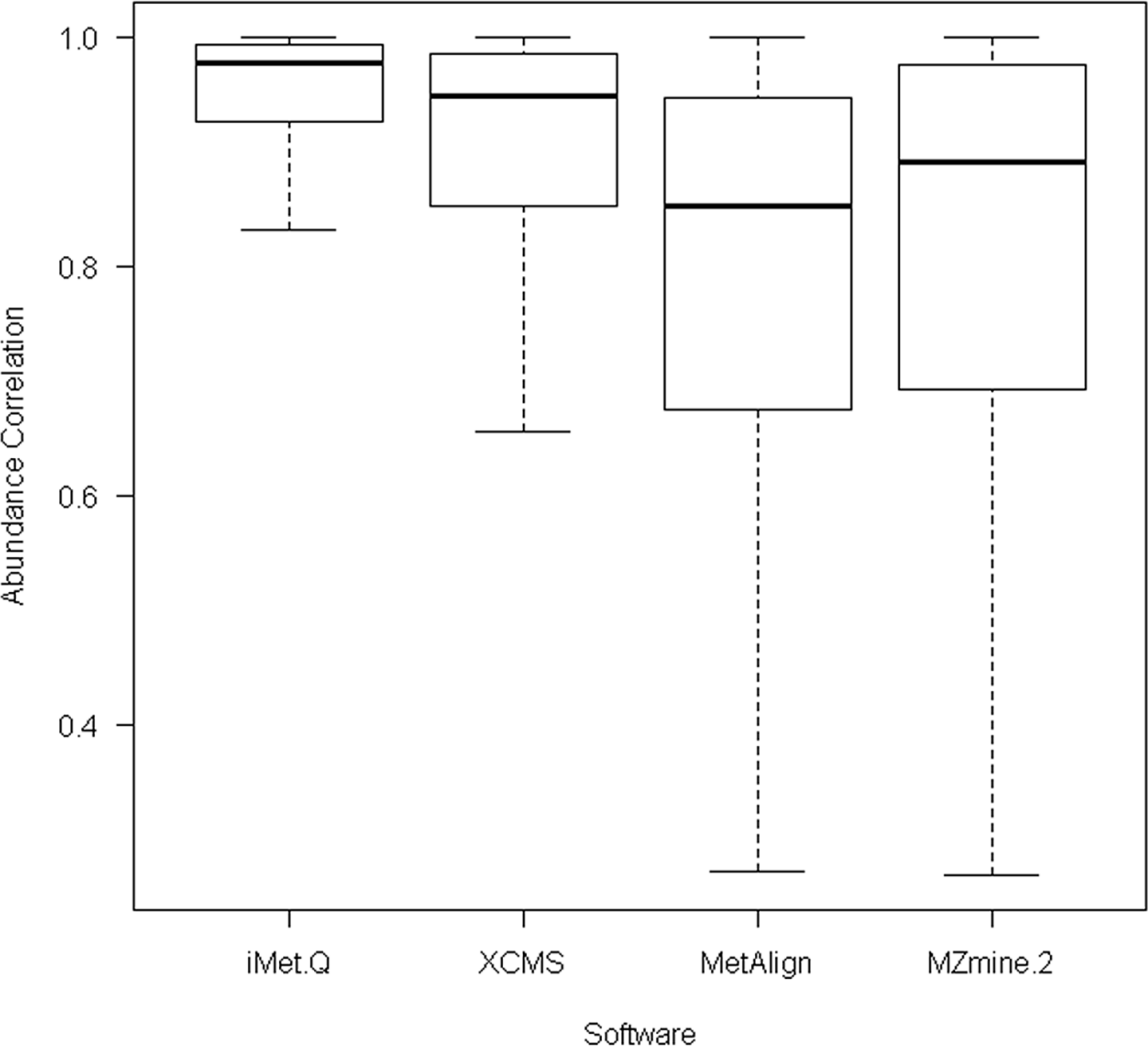
The box plot of abundance correlation of 167 elucidated metabolites across replicates in the public Arabidopsis data detected by the four quantitation tools.

For 167 elucidated metabolites, we searched their *m/z* and retention time values provided in [28] against the quantitation results of the four tools with m/z and retention time tolerances of 0.05 m/z and 9 seconds. As a result, iMet-Q detected all of the elucidated metabolites, whereas XCMS, MetAlign, and MZmine 2 detected 103 (62%), 145 (87%), and 129 (77%) of the elucidated metabolites, respectively. We further evaluated the detected abundance of these metabolites. Since the exact abundance of 167 elucidated metabolites was unknown, we calculated the abundance correlation between any two replicates of a sample by PPMCC. As a result, iMet-Q achieved an average replicate abundance correlation of 0.92, whereas XCMS, MetAlign, and MZmine 2 achieved average abundance correlations of 0.87, 0.78, and 0.81, respectively (as shown in Fig 3). The result revealed that iMet-Q not only achieved high accuracy on detecting the 167 elucidated metabolites but also provided very consistent quantitation result.

**Fig 4:**
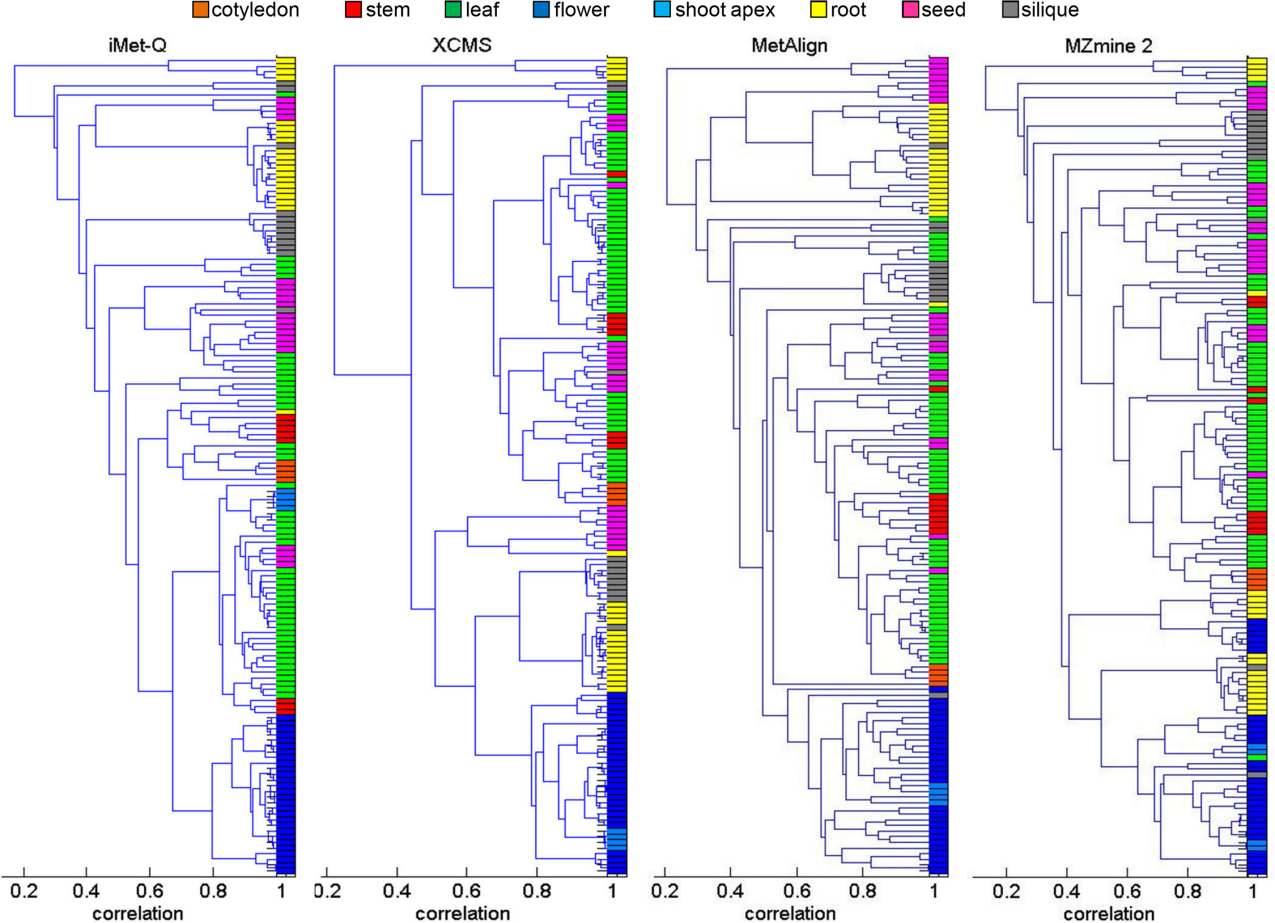
Hierarchical clustering by using the quantitation results of iMet-Q, XCMS, MetAlign, and MZmine 2. Each entry in the tree leaves of a dendrogram represents a replicate. For each tool, we first combined its quantitation results of positive- and negative-ion modes. Colors were assigned to each replicate in the combined quantitation results according to the plant classes which the replicates originated from as follows: orange for cotyledon, red for stem, green for leaf, blue for flower, light blue for shoot apex, yellow for root, pink for seed, and gray for silique. Next, the figure was produced using MATLAB *dendrogram* function with PMMCC as the abundance correlation measure between any two replicates in the combined quantitation results.

Next, we examined whether replicates of the 36 samples could be properly clustered into eight plant classes using the quantitation results of the four tools. We combined the quantitation results from positive- and negative-ion modes, and performed hierarchical clustering using MATLAB *dendrogram* function with PPMCC as the abundance correlation measure. The combined quantitation results of the four tools are provided in S7–S10 Tables. The numbers of detected peaks in the combined quantitation results of iMet-Q, XCMS, MetAlign, and MZmine 2 were 13079, 7487, 37364, and 19394, respectively. Fig 4 shows the hierarchical clustering results of the four tools. The average abundance correlations of the eight plant classes using the combined quantitation results of iMet-Q, XCMS, MetAlign, and MZmine 2 were 0.71, 0.75, 0.65, and 0.59, respectively. The detailed average abundance correlation of each plant class is shown in S11 Table. Note that all the replicates of flower class, the best clustered plant class (blue color in Fig 4), were clustered using iMet-Q’s combined quantitation result, and the average abundance correlation of the flower class was 0.88. This evaluation implies that, although the numbers of peaks detected by MetAlign and MZmine 2 were much larger than those detected by iMet-Q and XCMS, peaks detected by iMet-Q and XCMS might be more essential for distinguishing plant classes.

**Fig 5:**
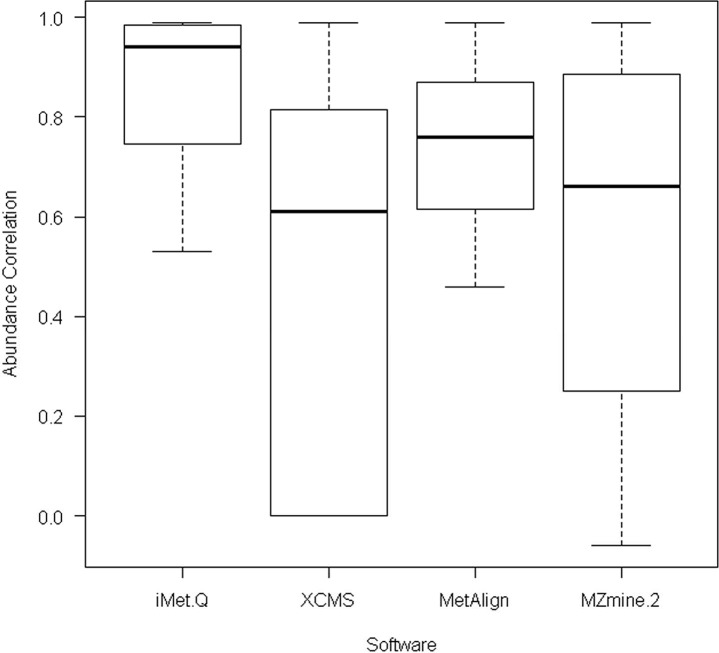
The box plot of abundance correlations between 19 verified metabolites and their in-source fragments detected in the public Arabidopsis data.

To overcome the bottleneck in metabolite identification based on MS1 data, a method utilizing both isotope ratio and in-source fragment information has been recently proposed, in which the same Arabidopsis metabolome data set was also used for identification performance evaluation [35]. According to the study, 19 out of 167 elucidated metabolites have been confirmed that some of their in-source fragments were detected in MS1 scans [35], and the abundance correlations between the 19 metabolites and their fragments were above 0.8. We thus used the 19 confirmed metabolites to demonstrate the ability of iMet-Q. By calculating the abundance correlations between 19 metabolites and their in-source fragments, iMet-Q reported an abundance correlation of 0.94, whereas XCMS, MetAlign, and MZmine 2 reported abundance correlations of 0.61, 0.76, and 0.66, respectively (as shown in Fig 5). This result revealed that the abundances of the 19 metabolites and their in-source fragments were accurately detected and calculated by iMet-Q. Furthermore, isotope ratios of 89.47% (17 out of 19) metabolites were accurately calculated by iMet-Q, with differences within 0.05 to the theoretical ratio (S12 Table).

### Friendly User Interfaces of iMet-Q

**Fig 6:**
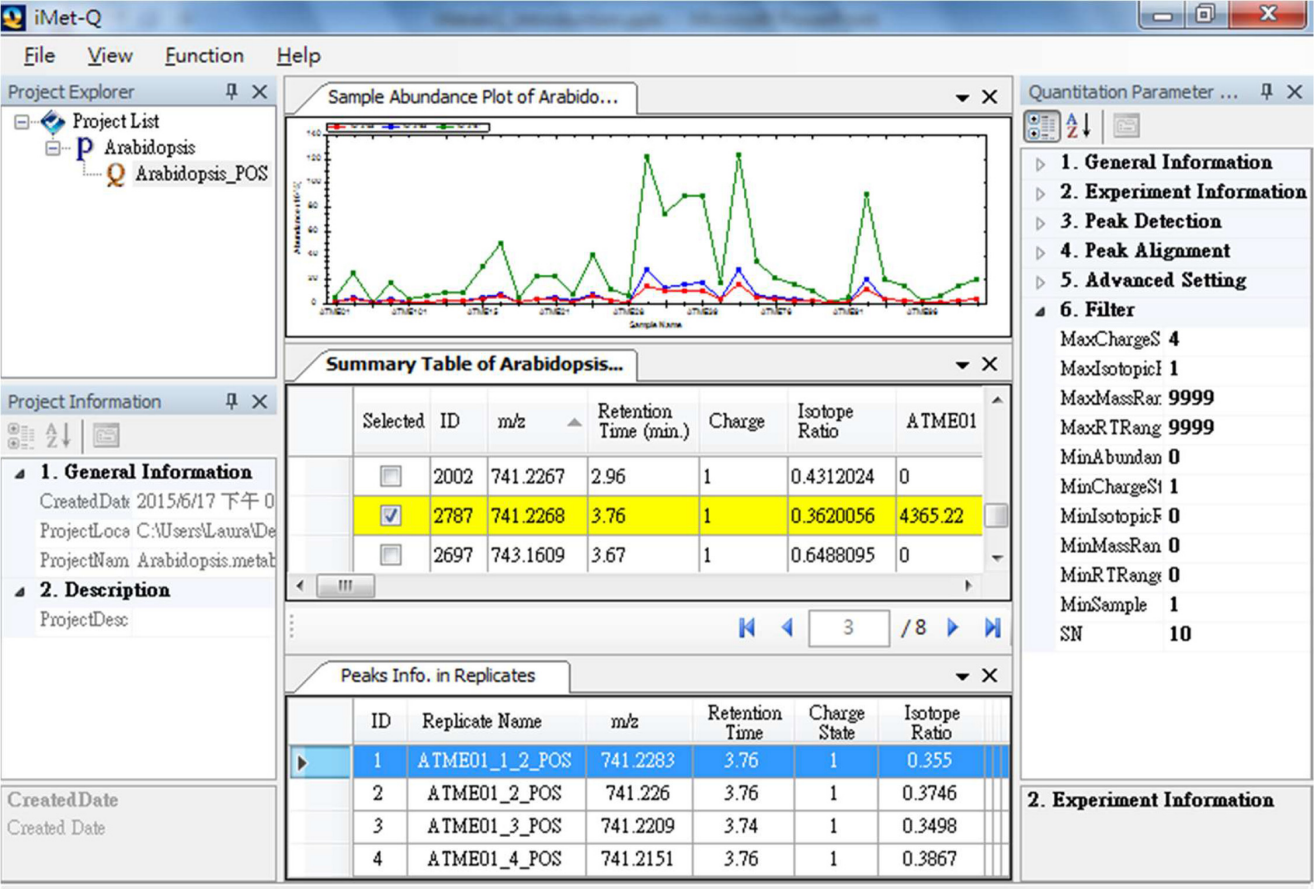
The main graphical user interface of Metab-Q. The Arabidopsis data from positive-ion mode is used as an example. After processing the data, Metab-Q lists the detected peaks in the summary table where the peaks are sorted according to their retention time. When users select peaks of interest in the summary table, the abundances of the selected peaks in different samples are plotted in the sample abundance plot and the detailed information of the selected peak in the technical replicates of a sample is listed in the panel below the summary table. The left panel is the quantitation parameter explorer that lists the parameters of a quantitation. Users can use the provided filter function to narrow down the number of peaks in the summary table.

We particularly implemented iMet-Q as a project-oriented quantitation platform, allowing users to conveniently quantify, manage and compare a large number of data sets or quantitation results in Microsoft Windows series platform (including Windows 7, 8, and Windows Server 2008, 2012). To be specific, iMet-Q applies a tree structure to organize quantitation results, where each node represents a project and sub-nodes of a project represent the quantitation results (as shown in Fig 6).

iMet-Q provides three wizards to guide users easily executing the program as follows. First, a *project wizard* guides a user to create a project and designate a folder to store the project. Second, a *quantitation wizard* guides the user to quantify LC-MS-based data. The wizard only requires the user to set up three quantitation parameters, i.e., mzWidth, mzTol, and rtTol; other parameters are optional. Since multiple technical replicates may be conducted on a sample, iMet-Q pops up a table to guide users to group technical replicates by the sample. During quantitation, iMet-Q sequentially detects peaks from each replicate and then aligns those detected peaks across replicates/samples. Third, an *export wizard* guides the user to export quantitation results in common output formats, including.csv and.txt. It also allows users to name the quantitation, designate the file for exporting, and set up criteria for filtering out unwanted peaks in order to focus on peaks of interest. With the conventional output formats, the user can easily import the quantitation results to other available tools, such as Microsoft Excel and Matlab, for further statistical analysis.

To compare abundances across samples, iMet-Q hierarchically displays a quantitation result in the main interface, where the quantitation result is summarized in a summary table. The summary table lists the *m/z*, retention time, charge state, isotope ratio, and abundance of all detected peaks in all samples for a quick overview. The user can click on a detected peak of interest in the summary table, and the sample abundances of the selected peak will be plotted in the upper panel above the summary table. Meanwhile, the detailed information of the selected peak in the technical replicates of a sample will be listed in the panel below the summary table. The user can double-click a row in the replicate table to view the extracted ion chromatogram of the peak. In order to efficiently select peaks of interest in the summary table, iMet-Q provides a search function using the range of *m/z*, retention time, charge state, or isotope ratio to narrow down the peaks listed in the summary table.

## Conclusions

Many state-of-the-art quantitation tools have been proposed to allow users automatically quantifying metabolites from large-scale label-free metabolomics data sets. The quantitation ability of these tools is beyond doubt. However, a major concern of using these tools is the parameter setting for peak detection. Since peak shapes vary greatly from one to another, it is difficult for users to select an optimized parameter setting for peak detection. In addition, most of the tools do not provide deisotoping, and thus users have to apply additional packages for isotope pattern assembly. Therefore, in this paper, we introduced an intelligent quantitation tool, iMet-Q, which is capable of dynamically determining the peak widths in liquid chromatogram dimension without input parameter, and automatically performing isotope pattern assembly. In our evaluation, iMet-Q has the quantitation performance better than XCMS, MetAlign, and MZmine 2 on standard and large-scale metabolome data sets. Besides, iMet-Q provides both the charge states and isotope ratios of detected peaks. With the charge state information, users can obtain accurate masses of detected peaks. Meanwhile, the isotope ratio information provides users an opportunity to reduce metabolite candidates. Although our evaluation demonstrates that the metabolite isotope ratios calculated by iMet-Q are close to the theoretical values, it is important to note that isotope ratio can be affected by noise interference, saturation effects and different experimental conditions [36, 37]. In addition to providing accurate quantitation, charge state, and isotope ratios, iMet-Q is equipped with friendly user interfaces so that quantifying metabolites become an easy task. The software program is now publicly available for download at http://ms.iis.sinica.edu.tw/comics/Software_iMet-Q.html.

## Supporting Information

S1 File The XCMS quantitation results of the standard metabolite mixture (profile mode) under five different parameter settings. (ZIP)

S2 File The XCMS quantitation results of the standard metabolite mixture (centroid mode) under 37 different parameter settings. (ZIP)

S3 File The XCMS quantitation results of the public Arabidopsis dataset (positive mode) under 37 different parameter settings. (ZIP)

S4 File The XCMS quantitation results of the public Arabidopsis dataset (negative mode) under 37 different parameter settings. (7Z)

S1 Section The detailed description of centroiding signals in each scan. (PDF)

S1 Table The sample ratio of each metabolite under different fwhm (for Standard Mixture-profile). (XLSX)

S2 Table The sample ratio of each metabolite under different centWave parameters (for Standard Mixture-centroid). (XLSX)

S3 Table The reproducibility and abundance consistency of the internal standard (lidocaine) in Arabidopsis data set (positive mode) under different centWave parameters. (XLSX)

S4 Table The reproducibility and abundance consistency of the internal standard (camphor-10-sulfonic acid) in Arabidopsis data set (negative mode) under different centWave parameters. (XLSX)

S5 Table The abundances of seven standard metabolites detected from profile and centroid data set using four quantitation tools. (XLSX)

S6 Table The number of metabolite candidates of the seven standard metabolites using (1) mass match, and (2) mass and isotope ratio (IR) match. (XLSX)

S7 Table The combined quantitation result generated using iMet-Q. (XLSX)

S8 Table The combined quantitation result generated using XCMS. (XLSX)

S9 Table The combined quantitation result generated using MetAlign. (XLSX)

S10 Table The combined quantitation result generated using MZmine 2. (XLSX)

S11 Table The average abundance correlations of the eight plant classes using the quantitation results obtained from iMet-Q, XCMS, MetAlign, and MZmine 2. (XLSX)

S12 Table The isotope ratio of the 19 identified metabolites in the public Arabidopsis data set. (XLSX)
