# Supplementary material for: iMet-Q: A User-Friendly Tool for Label-Free Metabolomics Quantitation Using Dynamic Peak-Width Determination
Source: PLoS One. 2016 Jan 19;11(1):e0146112. doi: 10.1371/journal.pone.0146112 (PMC4718670; doi:10.1371/journal.pone.0146112)
Supplement: S1 Section — (PDF) [file pone.0146112.s005.pdf]

Supporting Information

iMet-Q: a user-friendly tool for label-free metabolomics quantitation using dynamic peak-width determination

Hui-Yin Chang[1,2,3], Ching-Tai Chen[3], T. Mamie Lih[1,2,3], Ke-Shiuan Lynn[4],

Chiun-Gung Juo[5], Wen-Lian Hsu[3], Ting-Yi Sung[3,*]

[1] Bioinformatics Program, Taiwan International Graduate Program, Academia Sinica, Taipei 11529, Taiwan

[2] Institute of Biomedical Informatics, National Yang-Ming University, Taipei 11221, Taiwan

[3] Institute of Information Science, Academia Sinica, Taipei 11529, Taiwan

[4] Department of Mathematics, Fu Jen Catholic University, New Taipei City 24205, Taiwan

[5] Molecular Medicine Research Center, Chang Gung University, Taoyuan 33302, Taiwan

* Corresponding author
E-mail: tsung@iis.sinica.edu.tw (TYS)

S1 Section. The detailed description of centroiding signals in each scan

For profile-mode data, to centroid each survey (i.e., MS1) scan, iMet-Q starts from the rightmost (smallest *m/z*) signal of a scan as the seed to perform the procedure of *group-and-extend* for the entire scan as follows. By grouping, iMet-Q incrementally groups signals close to the seed within a user-defined *m/z* tolerance, *d* (i.e., mzWidth in iMet-Q). When encountering a signal with higher intensity than the seed, extend the group by updating the higher-intensity signal as the seed to further perform grouping until no signal with higher intensity than the seed can be found. After obtaining an extended signal group, if the group width, defined as the *m/z* difference of the first signal and the last signal in the group, is no larger than *d*, the highest-intensity signal is selected to represent the signals in the group. Otherwise, iMet-Q splits the group into several signal groups as follows. Starting from the highest-intensity signal in the group, iMet-Q reaches out for the signals that are within $\pm d$ and with decreasing intensities. These signals are clustered to form a new group. Then the highest-intensity signal in the new group is selected as the representative and the rest are removed from further processing. The splitting procedure is repeated until all signals in the original group are processed. Then all of centroided signals will go through the subsequent procedures.
